# Remarks Made at a Clinic

**Published:** 1885-01-20

**Authors:** R. C. Word

**Affiliations:** Professor of Physiology in the Southern Medical College, Atlanta, Georgia


### REMARKS MADE AT A CLINIC.

By R. C. WORD, M. D., Professor of Physiology in the SOUTHERN MEDICAL COLLEGE, Atlanta, Georgia.

### VERATRUM—GELSEMIUM—ACONITE.

As these agents seem to be coming into general use, it is well to inquire into their destinctive properties.

Many practitioners seem to regard them as possessing the same therapeutic powers, and not infrequently they are combined in the same prescription ; and yet their physiological effects are somewhat different, and should be well studied in order that you may avail yourselves of those nice distinctions by which careful and judicious practitioners so often excel in the administration of drugs.

*Veratrum veride.*—There has been much discussion in regard to the physiological action of Veratrum veride, and its real *modus operandi* is not yet fully understood ; but the most probable view is that it acts directly upon the pneumogastric nerve. This nerve, you are aware, sends branches to the great cardiac plexus and to all the viscera of the trunk and abdomen. It is a nerve which exercises most extensive and important influences, being excito-motory, excito-secretory, and excito-nutrient. Any agent, therefore, which acts specifically upon this nerve, or upon the medulla oblongata, the point of its organ, must possess great power for good or evil.

It is known that the pneumogastric has an inhibitory influence upon the heart's action. A galvanic current passed through the pneumogastric diminishes the frequency of the pulsations.

Whatever be the method of its action, veratrum certainly does influence and control, in a most remarkable manner, the frequency of the heart's action. It also lessens the frequency of the respiratory movements, and, if given to excess, may even lead to asphyxia. Veratrum is called an arterial sedative, and a depressant of great and dangerous power ; on which account many regard it as a dangerous remedy. Nearly all who have written on the subject caution the practitioner against its use in low and feeble states of the circulation. I do not regard it so dangerous an agent in low states of the system as many who have thus written upon it. When first introduced by Dr. Norwood, of South Carolina, as a remedy in pneumonia, it was prescribed in eight-drop doses of the saturated tincture, gradually increased, and at intervals of three hours. Experience soon showed that the dose was excessive, and the severe vomiting and prostration which often resulted alarmed the profession and greatly disparaged the remedy for a time. I have, during an experience of more than thirty years in the practice of medicine, very largely, and successfully, relied upon veratrum in the early stages of pheumonia, and I have found that, when properly administered, nausea and depressing influences do not result from its use.

I usually commence the remedy in one or two drop doses at intervals of two hours, increasing a drop with each dose, until the desired impression upon the heart's action is obtained ; after which the dose is not increased, or is diminished, according to circumstances. I dilute the dose in at least two tablespoonsful of water, or instruct the patient to swallow a wineglass full of water after taking the medicine, to avoid the irritating effects of the drug upon the mucous membrane of the stomach. I also, especially if there be pain the case, or any tendency to diarrhœa, add to the dose a few drops of *Elix. opii* or a little paregoric. The opiate counteracts the nauseating tendency of the drug, but does not interfere with its action upon the circulation. When using veratrum, it is not deemed advisable to give quinine or other agent, except the opium in the manner mentioned.

Veratrum may be used in any case of an inflammatory or sthenic character, where it is desired to lessen the frequency of the pulsations, and thus reduce the fever. In scarlatina its results have been very satisfactory. Its influence upon the nervous system has also proven available for the relief of puerperal convulsions, when used in decided doses. When veratrum has been long continued at a dose sufficient to keep the pulse at a given point, and then continued in smaller doses as the patient improves, it is not cumulative, but acts as a tonic, increases the appetite and invigorates the system.

*Gelsemium.*—Gelsemium, or the yellow jessamine, exerts an influence upon the cerebro-spinal nerve centres, producing a soothing and slightly hypnotic effect upon both the motor and sensory nerves throughout the system. It lessens, to some extent, the heart's action, and reduces fever, by its tranquilizing effect upon the general nervous system. It does not slow the pulse-rate so greatly as veratrum, but there is a greater lowering of the temperature relative to its action upon the heart than with veratrum. It has the property of lowering the tone, and relaxing unstriped muscular fibre ; hence it is a good remedy to relax rigid os uteri and to facilitate the first stage of labor. In convulsions, or in any condition dependent upon exaltation of the motor and sensitive spheres, it is a good remedy. Unlike veratrum, it does not produce nausea or emesis, but is rather soothing in its influence upon the mucous membranes. In irritable bladder, or incontinence of urine, this soothing effect upon the mucous membranes is well marked, especially when combined with bromide of potassium, as in the following formula:

| | | |
|---|---|---|
| ℞. | Brom. potassium | ℥ ss. |
| | Water | ℥ iij. |
| | Tinct. gelsemium | ʒ ii. |

M. Take teaspoonful every three to four hours.

In high fibrile constitutions, attended with headache, from hyperæmia of the brain, it usually has a quieting influence, reducing, in some degree, the frequency and tension of the pulse, and lowering the temperature. I have sometimes combined it with the spirits of nitre and veratrum with good effect in such conditions:

| | | |
|---|---|---|
| ℞. | Tinct. gelsemium | ʒ i. |
| | Tinct. veratrum | gtt. xxiv. |
| | Spirits nire dulc | ʒ ii. |
| | Water | ℥ iij. |

M. Teaspoonful every hour until the fever subsides, and then lengthen the interval.

I have found it useful in neuralgia, combined with bromide of potassium and opium, as follows:

| | | |
|---|---|---|
| ℞. | Tinct gelsemium | gtt. xv. |
| | Elix. opium | gtt. xx. |
| | Brom. potas | gr. xxx. |

M. This given at a single dose rarely fails to relieve the pain and give rest to the patient. May repeat after two hours, if necessary.

In ordinary toothache, twelve to fifteen drops of gelsemium gives relief for a time. The tendency of opium to determine to the brain seems to be counteracted by gelsemium, in some measure at least, and yet its anodyne effect is promoted rather than diminished when combined with gelsemium. I have noticed its good effect, especially in that annoying form of toothache that makes its attack immediately after going to sleep, or when the patient gets warm in bed.

In ovarian neuralgia, and in palpitation of the heart, connected with, or dependent upon, ovarian or uterine disorders, it has, usually, a quieting effect, especially if combined with the bromides.

*Aconite.*—This agent is the sheet-anchor of the homœpaths, and to it, more than to any other agent, is due their vaunted success in the treatment of febrile and inflammatory affections. Aconite affects the sensory nerves rather than the motor. In toxic doses it paralyzes, first, the peripheral termination, then the nerve centers, and, lastly, the centers of sensation in the cord. In proper doses, it lessens the number of heart-beats, not so much or so decidedly as veratrum, but reduces the temperature more in proportion than either veratrum or gelsemium. It is a more powerful and dangerous agent than either of the others which I have mentioned. It is sedative to the vasso-motor nerves, and without so strongly impressing the pneumogastric as veratrum; it yet slows the respiration, and tranquilizes the general nervous system. The eliminating powers of aconite are superior to either veratrum or gelsemium; especially is this true of its influence upon the skin and kidneys.

It opposes the fever process by lowering the temperature and by slowing respiration. These properties give to the remedy remarkable antiphlogistic powers, and adapt it to all inflammatory conditions. In severe attacks of neuralgia it is more reliable and decided than gelsemium, and, combined with opium and bromide, in place of gelsemium, in the formula given under that head, it is equally, if not more, efficient in giving relief. The dose is, of course, less, and sometimes acts very efficiently without the opium, as follows:

℞. Tinct. aconite.............................. gtt. iv.
Brom. potash.............................. grs. xxx.

Give at a dose, dissolved in water, and repeat in one hour if necessary, using revulsives, also to the affected part. This formula is adapted to the severer forms of neuralgia, especially of the facial variety.

In convulsive affections with fever, aconite is useful, but, as a rule, less powerful in diminishing motor activity than gelsemium. In throat inflammations, particularly in tonsilitis, it is a good remedy. In pneumonia it is also a useful remedy, and is preferred in cases of children, and that class of cases where, from anæmia, debility or like cause, the veratrum or gelsemium is contraindicated. It may be given even in cases attended with considerable prostration, and in cases where the pulse is small and somewhat feeble, especially if there be inflammatory action at any point. In gastric inflammation it does not act well, its tendency being to produce nausea.

Aconite should be given in small doses oft repeated. As a febrifuge, given during the exascerbation of our malarial fevers, it is excellent. My method of using it in these, and in most cases of febrile excitement, is as follows:

℞ Tinct. aconite radix.......................... gtt. v.
Water.................. .................... ℥ iv.

M. Teaspoonful every half hour until fever begins to abate, and then at longer intervals, the patient being instructed to discontinue it if the fever goes off, or to suspend the remedy if sweating supervenes. This formula will usually suit children over five or six years old. If younger, the dose should be lessened. In high fevers of adults I often add ten or twelve drops to the four ounces of water. Spirits of nitre may be advantageously added, especially where the urine is red and scant :

℞. Tinct. aconite............................. ... gtt. x.

Spirits nitre.................................... ʒ j.

Water.......... ............................ ℥ iv. M.

Teaspoonful every half to one hour until fever abates. Though the fever may not entirely subside, under this remedy, it always diminishes more or less, the temperature is lowered, the headache is palliated, and the exasperation is thus passed over without untoward results, and with comparative comfort to the patient.

